# Distinct age‐adjusted D‐dimer threshold to rule out acute pulmonary embolism in outpatients and inpatients

**DOI:** 10.1111/crj.13728

**Published:** 2024-02-11

**Authors:** Peng Liu, Haixu Yu, Wei Liu, Lin Lin, Ying Qun Ji

**Affiliations:** ^1^ Department of Heart Intensive Care Unit The First Affiliated Hospital of Dalian Medical University Dalian China; ^2^ Department of Cardiology, Beijing Jishuitan Hospital Capital Medical University Beijing China; ^3^ Department of Clinical Laboratory The First Affiliated Hospital of Dalian Medical University Dalian China; ^4^ Department of Pulmonary and Critical Care Medicine, East Hospital Tongji University Shanghai China

**Keywords:** acute pulmonary embolism, age‐adjusted, cutoff level, D‐dimer, diagnosis

## Abstract

**Introduction:**

The diagnosis of acute pulmonary embolism (PE) is combinations of clinical probability assessments, plasma D‐dimer (DD) test results, and/or computed tomographic pulmonary angiography (CTPA).

**Objective:**

The aim of this study is to explore the appropriate DD cutoff using the immunoturbidimetric method in outpatients and inpatients.

**Methods:**

We retrospectively enrolled 2689 patients with suspected PE between January 2014 and December 2019. All patients underwent clinical probability assessments, DD tests, and CTPA. We investigated the appropriate cutoff level for plasma DD tests in the correlation analysis and receiver operating characteristic (ROC) curves.

**Results:**

Among all patients, 1263 were confirmed acute PE. The age‐adjusted DD level was determined to be age × 10 μg/L (for patients aged >50 years) in outpatients. This cutoff value resulted in a sensitivity of 96.75% and a specificity of 87.02%, with the area under the curve (AUC) of 0.908 and the number needed to treat (NNT) of 1.18. For inpatients, the age‐adjusted cutoff values for the biomarker DD demonstrated poor specificity (13.34%) and NNT (9.88). However, when the DD cutoff was adjusted to 2 × the upper limit of normal (ULN), the sensitivity increased to 93.19%, while the specificity remained at 29.55%, with the AUC of 0.610 and the NNT of 4.76. The optimal DD cut‐off value was 3010 μg/L (about 5 × ULN), resulting in a sensitivity of 75.22% and specificity of 61.72%, with the AUC of 0.727 and the NNT of 2.7.

**Conclusion:**

Using the immunoturbidimetric method to measure DD, an age‐adjusted DD cutoff (age × 10 μg/L, if aged >50 years) should be considered for outpatients with suspected PE. For inpatients, increasing the DD cutoff value to at least 2 × ULN yields the best test performance.

## Graphical abstract

Using the immunoturbidimetric method to measure D‐dimer, an age‐adjusted D‐dimer cutoff (age×10μg/L, if aged >50 years) should be considered for outpatients with suspected PE. But for inpatients, increasing the D‐dimer cutoff value to at least 2×ULN yields. It may reduce the unnecessary CTPAs in older patients.

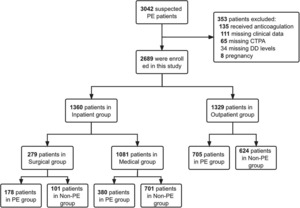

## 1 INTRODUCTION

After myocardial infarction and stroke, acute pulmonary embolism (PE) is the third most common cause of cardiovascular death. The annual incidence of PE is 39–115 per 100 000 population in Europe and the United States.^1^, ^2^ The clinical manifestations of acute PE often include chest pain, sudden shortness of breath, and syncope, but may also manifesting non‐specific symptoms. D‐dimer (DD) is a protein fragment formed by the degradation of blood clots that is the most commonly used thrombotic biomarker in the clinical workup. The diagnostic value of DD is characterized by a high negative predictive value with low positive predictive value.^3^ Several studies suggest that using a combination of a normal DD value and low clinical probability as diagnostic criteria can help to rule out PE in 30% of outpatients with suspected PE without the need for further examinations.^4^, ^5^

In general, DD levels increase with age, and older individuals are more likely to receive a false positive DD result when using a standard DD cutoff value. Increasing the specificity of the DD test in the elderly may reduce the risk of radiation exposure, contrast‐induced nephropathy, hospital admission rates, and excessive medical costs.^3^ Hospital‐acquired PE remains one of the most common etiologies, accounting for more than one‐third of all diagnosed venous thromboembolism (VTE).^6^ Besides the age, DD levels are influenced by other conditions such as cancer, inflammatory diseases, pregnancy, surgery, trauma, and so forth. Therefore, the diagnostic efficacy of DD testing needs to be improved, especially for inpatients with multiple comorbidities. Hence, the 2019 European guideline on acute PE recommended the use of age‐adjusted DD cutoff value (age × 10 μg/L) for suspected PE patients aged >50 years with low or intermediate‐clinical probability (Class of recommendation IIa, Level of evidence B).^7^, ^8^ The currently international plasma DD cutoff value is based mainly on studies predominately conducted in White population from the Europe and the United States, even though previous studies have suggested that ethnicity plays a role in PE and should be considered in the pre‐test probability assessment of patients with suspected PE. Furthermore, the vast majority of studies on age‐adjusted DD cutoff levels have used DD values measured using enzyme‐linked immunosorbent assay (ELISA).^4^, ^7^, ^8^ Although ELISA is the most prevalent method for detecting plasma DD worldwide, other available test methods, such as the latex‐enhanced immunoturbidimetric assays, are more common used in China, the world's most considerable low‐ and middle‐income country.^9^, ^10^ However, limited studies regarding the alternative DD cutoff value measured using the immunoturbidimetric assay in the Chinese population. Therefore, our study aimed to determine the optimal DD cutoff level using the immunoturbidimetric assay for both Chinese outpatients and inpatients with suspected PE.

## 2 MATERIALS AND METHODS

### 2.1 Study populations and design

**FIGURE 1:**
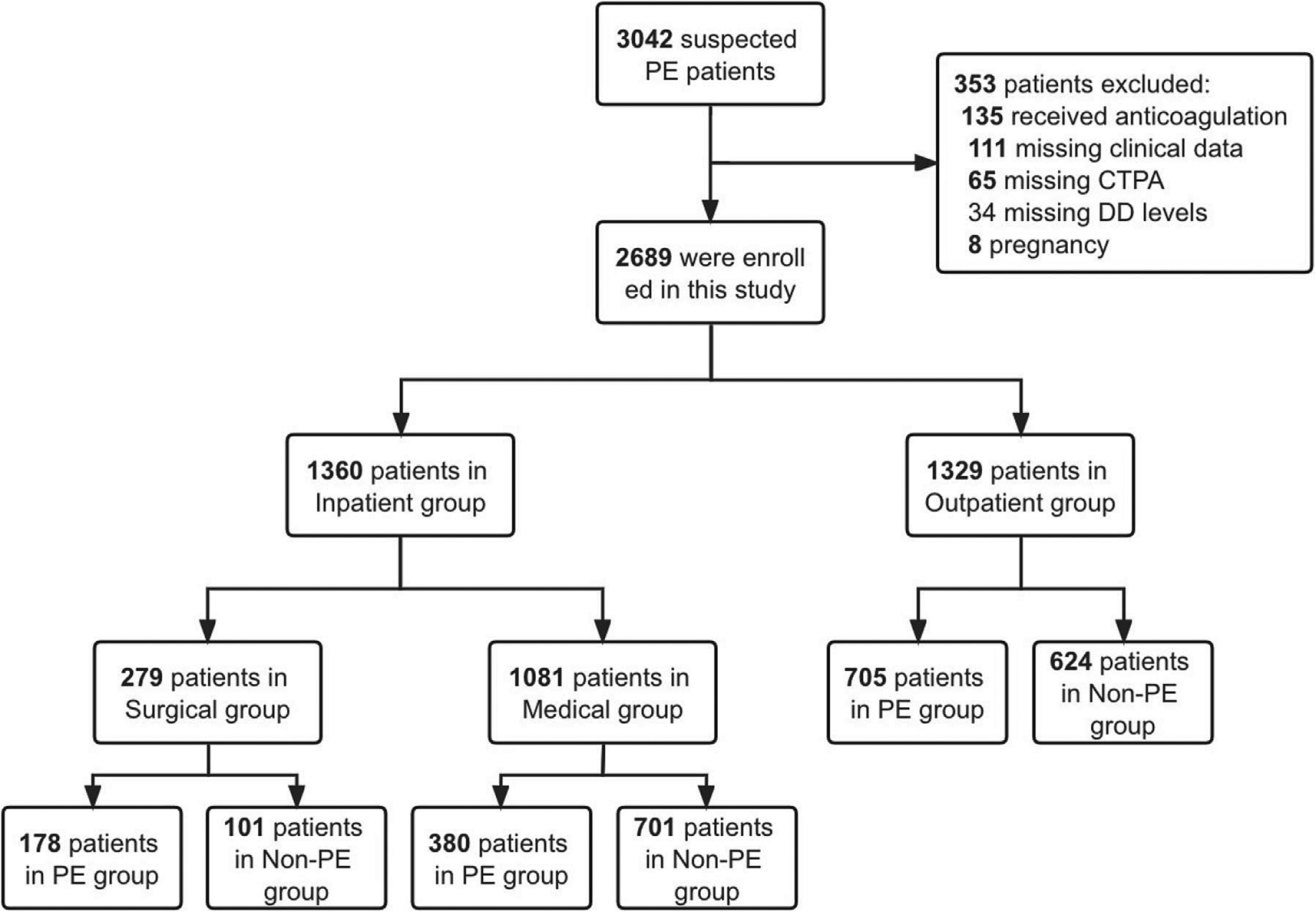
Flow diagram of the study. CTPA, computed tomographic pulmonary angiography; PE, pulmonary embolism.

In this retrospective study, consecutive patients with clinically suspected PE were recruited between January 2014 and December 2019 from the First Affiliated Hospital of Dalian Medical University. According to the amended Declaration of Helsinki, this study protocol was approved by the ethics committee of the First Affiliated Hospital of Dalian Medical University, and all patients gave written informed consent (LCKY2017‐17). Clinical suspicion of PE defined as the presence of any of the following: unexplainable dyspnea, chest pain, hemoptysis, syncope, palpitation, cyanosis, asymmetric swelling of lower limbs, or previous history of VTE; exclusion criteria are as follows: (1) PE suspicion was raised more than 24 h after admission to the hospital in outpatient and less than 2 days of hospitalization in inpatient; (2) Aged <18 years old; (3) PE was suspected, but the patient received anticoagulation therapy for other reasons (such as atrial fibrillation); (4) missing data (CTPA, DD, or clinical data); (5) ongoing pregnancy (shown in Figure 1).^3^, ^11^ Clinical probability was assessed using the simplified Wells score, and CTPA was performed as the gold standard test for PE. Patients were divided into PE and non‐PE groups based on the CTPA results and into inpatient and outpatient groups based on the source of recruitment.

#### 2.1.1 DD test

Plasma DD levels were measured using the INNOVANCE® D‐dimer based on the SYSMEX cs5100 system (Siemens Healthcare Diagnostics, Erlangen, Germany) according to the manufacturer's instructions.^10^ The normal range for DD was defined as 0–550 μg/L. The intra‐assay and inter‐assay coefficients of variation for DD were <15%.

#### 2.1.2 CTPA

The GE Revolution 64‐slice CT scanner (General Electric, WI, USA) was used to acquire images of the thorax after intravenous injection of the contrast agent. Patients received 20–30 mL of contrast agent (300 mg/mL) injected into an antecubital vein at a rate of 3.5–4.0 mL/s using an automated injection system. The following CT parameters were used: tube current 400–500 mA; tube voltage 120 kV–140 kV and slice acquisition 512 × 512 mm. Image slices of the entire thorax were reconstructed with 1.5 mm thickness in a transverse orientation. PE was diagnosed if at least one intraluminal filling defect was identified in the pulmonary artery tree. The CTPA scans were independently assessed by two blinded expert thoracic radiologists. The consensus was reached through discussions in the event of disagreements.

#### 2.1.3 Simplified Wells score

The simplified Wells score was calculated based on the criteria.^7^ The clinical probability of PE was estimated using the Simplified Wells score for all patients. Scores ranged from 0 to a maximum of 7 points. Those with 0–1 points is defined as low clinical probability of PE; ≥2 points is defined as high clinical probability of PE.

### 2.2 Statistical analysis

All data were processed using SPSS 22.0 software (SPSS, Chicago, IL, USA). Data that were not normally distributed were expressed as a median and interquartile range [M (P25, P75)]. Continuous variables were compared using the Mann–Whitney *U* test (non‐normally distributed data). Count data were compared using the chi‐square test. Two‐tailed *P* < 0.05 was considered statistically significant. To determine the DD cutoff value, patients aged above 50 in the derivation set were divided into 10‐year age groups. The age‐DD equation was obtained from the correlation analysis. The equation coefficient was used as the age coefficient in the calculation of the age‐adjusted DD cutoff.^4^ Receiver operating characteristic (ROC) curves were constructed for each age group by Medcalc software version 19.2 (MedCalc Software Ltd, Ostend, Belgium). The sensitivity (SE), specificity (SP), positive predictive value (PPV), and negative predictive value (NPV) were computed. The number needed to treat (NNT) tested by D‐dimer to rule out pulmonary embolism was computed as 1 divided by the proportion of patients with a negative DD result in each age group.^8^ In the inpatient group and its subgroups, the ROC curve for the relationship between PE and DD was constructed to determine the Youden index and the best cutoff value.^12^, ^13^

## 3 RESULTS

### 3.1 Baseline characteristics of the study population

Among the 3042 suspected PE patients included, 353 patients were excluded from the study by exclusion criteria. A total of 1263 patients (47.0%) were confirmed PE (PE group), and 1426 (53.0%) were excluded diagnosis of PE (Non‐PE group). No significant differences in age and gender were observed between PE and Non‐PE group. The patients with PE had significantly higher simplified Wells scores and DD levels, and more often had prior VTE/PE, and immobilization or surgery in the past 4 weeks compared to patients without PE. Compared to outpatient group, patients with PE were associated with more DVT and thrombus load for inpatient group (Table 1).

**TABLE 1:** Baseline characteristics of included patients.

	Outpatient (1329)			Inpatient (1360)		
	PE group (705)	No‐PE group (624)	*P* value	PE group (558)	No‐PE group (802)	*P* value
Age (mean ± SD)	68.40 ± 13.65	68.99 ± 13.57	0.430	67.73 ± 12.87	68.61 ± 12.46	0.205
Male (*n*, %)	367 (52.0)	301 (48.2)	0.164	302 (54.1)	410 (51.2)	0.275
Hemoptysis (*n*, %)	44 (6.2)	30 (4.8)	0.255	35 (6.3)	46 (5.7)	0.680
Active cancer (*n*, %)	33 (4.7)	19 (3.0)	0.124	187 (33.5)	243 (30.2)	0.210
Prior VTE (*n*, %)	77 (10.9)	17 (2.7)	<0.001	20 (3.5)	15 (1.9)	0.049
Tobacco use (*n*, %)	277 (39.2)	221 (35.4)	0.145	228 (40.9)	338 (42.1)	0.636
Immobilization/surgery (*n*, %)	91 (12.9)	8 (1.3)	<0.001	283 (50.7)	170 (21.2)	<0.001
Comorbidities (*n*, %)						
DVT	472 (66.9)**	0 (0.0)	<0.001	398 (71.3)**	0 (0.0)	<0.001
Hypertension	175 (24.8)	134 (21.5)	0.149	123 (22.0)	172 (21.4)	0.792
Chronic kidney disease	28 (3,9)	24 (3.8)	0.906	32 (5.7)	40 (5.0)	0.544
Coronary artery disease	51 (7.2)	60 (9.6)	0.117	67 (12.0)	83 (10.3)	0.337
Congestive heart failure	28 (3.9)	29 (4.7)	0.543	43 (7.7)	39 (4.9)	0.030
COPD	27 (3.8)	16 (2.6)	0.193	55 (9.9)	37(4.6)	0.002
Diabetes	163 (23.1)	125 (20.0)	0.172	139 (24.9)	177 (22.0)	0.222
Atrial fibrillation	77 (10.9)	59 (9.4)	0.378	86 (15.4)	102 (12.7)	0.156
Clinical characteristic (Mean ± SD)						
HR at admission (bpm)	83.18 ± 16.37	80.55 ± 16.16	0.003	78.62 ± 13.08	74.68 ± 13.29	<0.001
SBP at admission (mmHg)	139.33 ± 23.50	137.59 ± 20.93	0.157	130.01 ± 19.88	133.63 ± 18.21	<0.001
SaO2 (%)	95.30 ± 2.11	95.07 ± 2.29	0.057	94.55 ± 2.07	95.02 ± 2.23	<0.001
Biological data						
Positive Troponin‐I (*n*, %)	69 (9.8)	77 (12.3)	0.137	167 (29.9)	106 (13.2)	<0.001
Positive BNP (*n*, %)	58 (8.2)	53 (8.4)	0.860	183 (32.8)	179 (22.3)	<0.001
D‐dimer (μg/L)	3790 (1680, 6055)**	270 (160, 440)**	<0.001	5495 (3028, 13 635)**	2090 (1000, 4508)**	<0.001
Simplified Wells score	2 (1,2)	0 (0,1)	<0.001	2 (1,3)	1 (0,1)	<0.001
CTPA findings (*n*, %)						
Saddle	17 (2.5)**	0 (0.0)	<0.001	157 (28.1)**	0 (0.0)	<0.001
Bilateral	222 (31.5)**	0 (0.0)	<0.001	299 (53.6)**	0 (0.0)	<0.001
Unilateral right/left	466 (66.0)**	0 (0.0)	<0.001	102 (18.3)**	0 (0.0)	<0.001
Treatment at discharge (*n*, %)						
DOAC	389 (55.1)	77 (12.3)	<0.001	377 (67.6)	133 (16.6)	<0.001
VKA	231 (32.8)	29 (4.6)	<0.001	129 (23.1)	52 (6.5)	<0.001
LMWH	67 (9.5)	5 (0.8)	<0.001	47 (8.4)	9 (1.1)	<0.001
None	18 (2.6)	513 (82.2)	<0.001	5 (0.9)	608 (75.8)	<0.001

*Note*: Variables are presented as median (IQR) or numbers (%).

Abbreviations: bpm, beats per minute; COPD, chronic obstructive pulmonary disease; DD, D‐dimer; DOAC, direct oral anticoagulant; DVT, deep vein thrombosis; HR, heartrate; LMWH, low‐molecular weight heparin; PE, pulmonary embolism; RV, right ventricle; SBP, systolic blood pressure; SpO_2_, oxygen saturation; VKA, vitamin K antagonist; VTE, venous thromboembolism.

** *P* < 0.001.

### 3.2 The optimal DD cutoff level among in outpatient group

**FIGURE 2:**
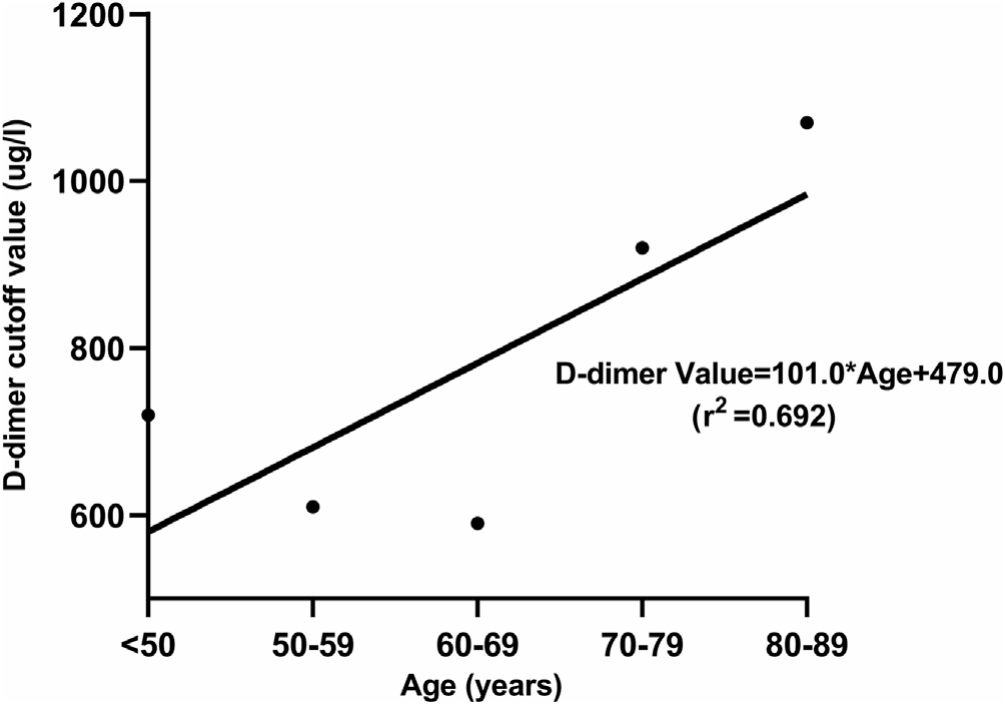
The figure showing optimal cut‐off values for DD test for pulmonary embolism by age in outpatient.

Depending on the source of recruitment, patients were divided into two groups: outpatients (1329 patients) and inpatients (1360 patients). Plasma DD levels and age were positively correlated in the outpatient group. The linear equation was Y = 101.0X + 479.0 (R^2^ = 0.692), and the equation coefficient was approximately 10.0. The age‐adjusted DD cutoff values in Chinese outpatients were in accordance with those recommended by the latest guideline (age × 10 μg/L, for patients aged >50 years) (Figure 2). Compared to the conventional DD cutoff value, age‐adjusted cutoff values significantly increased diagnostic efficacy with a sensitivity of 96.75% and a specificity of 87.02% in outpatient group (AUC = 0.908, *P* = 0.023, shown in Table 2).

**TABLE 2:** Comparison of different D‐dimer cutoff values and scores for all patients.

Cutoffs and scores (95%CI)	SE, % (95%CI)	SP, % (95%CI)	PPV, % (95%CI)	NPV, % (95%CI)	AUC (95%CI)	NNT (95%CI)
Conventional DD	97.73 (92.6–98.6)	83.33 (80.1–86.1)	86.9 (84.2–89.1)	97.0 (95.0–98.2)	0.905 (0.887–0.924)	1.20^a^
	98.56 (97.0–99.3)	7.60 (5.9–9.7)	42.6 (39.8–45.3)	88.4 (77.8–94.5)	0.530 (0.500–0.562)	16.20^b^
Age‐adjusted DD	96.75 (92.8–98.3)	87.02 (84.1–89.6)	89.2 (86.6–91.2)	96.9 (93.2–98.4)	0.908 (0.892–0.924)	1.18^a^
	96.77 (94.9–98.1)	13.34 (11.1–15.9)	43.7 (40.9–46.5)	85.6 (77.9–91.0)	0.551 (0.524–0.577)	9.88^b^
Simplified Wells score	60.00 (56.3–63.6)	94.71 (92.7–96.3)	92.8 (89.8–94.8)	67.7 (64.4–70.7)	0.773 (0.750–0.796)	1.65^a^
	61.65 (57.5–65.7)	77.93 (74.9–80.8)	66.0 (61.7–70.0)	74.5 (71.3–77.3)	0.698 (0.673–0.722)	2.52^b^

Abbreviations: DD, D‐dimer; NNT, number needed to treat; NPV, negative predictive value; PPV, positive predictive value; SE, sensitivity; SP, specificity.

^a^ Outpatient group.

^b^ Inpatient group.

### 3.3 The optimal DD cutoff level among in inpatient group

**FIGURE 3:**
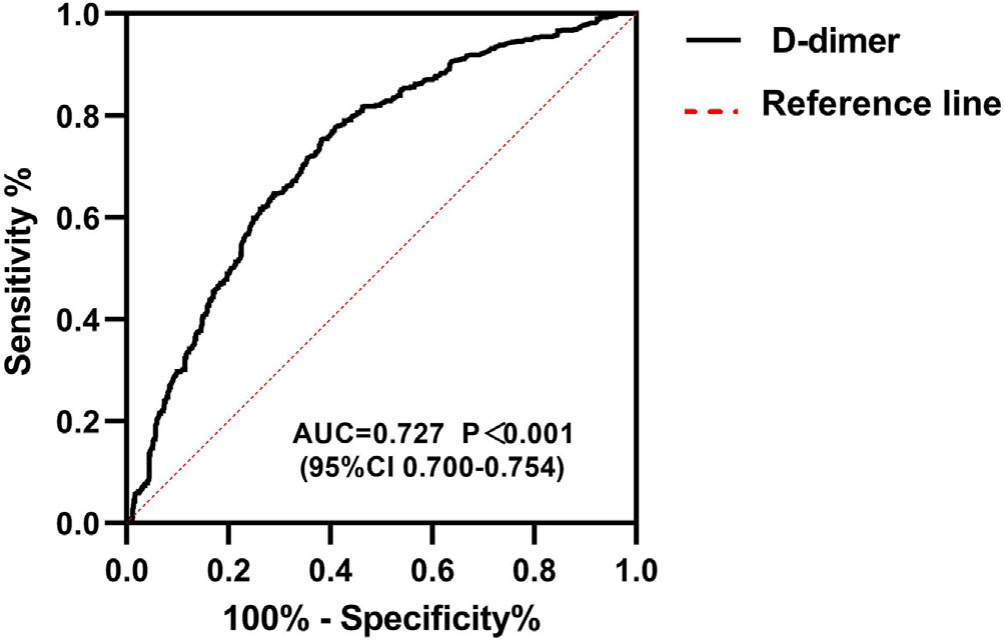
Results from ROC (receiver operating characteristics) analysis for the detection of pulmonary embolism from D‐dimer testing in inpatient.

For the inpatient group, we calculated the optimal DD cut‐off value, which was about 5.5 times higher than the upper limit of normal (ULN) (Figure 3). Nevertheless, the conventional DD cutoff value and the age‐adjusted cutoff value were insufficient to provide adequate diagnostic efficacy in the inpatients (AUC: 0.530 vs. 0.551, *P* < 0.001; NNT: 16.20 vs. 9.88, *P* < 0.001) (Tables 2 and 3). Rising from the conventional DD cutoff to 2 × ULN, the NNT was shifted from 16.20 to 4.76 (*P* < 0.001). When the DD cutoff was identified as 5.5 × ULN, the NNT was transformed from 4.76 to 2.76, and the diagnostic efficacy of AUC was statistical significance (2 × ULN vs. 5.5 × ULN, 0.610 vs. 0.727, *P* < 0.001), shown in Table 3).

**TABLE 3:** Comparison of different D‐dimer cutoff values for the inpatients.

Cutoffs	SE, % (95%CI)	SP, % (95%CI)	PPV, % (95%CI)	NPV, % (95%CI)	AUC (95%CI)	NNT (95%CI)
Conventional DD	98.56 (97.0–99.3)	7.60 (5.9–9.7)	42.6 (39.8–45.3)	88.4 (77.8–94.5)	0.530 (0.500–0.562)	16.20
2 × ULN	93.19 (89.8–94.4)	29.55 (26.4–32.8)	47.7 (44.7–50.7)	85.4 (80.0–88.8)	0.610 (0.584–0.636)	4.76
3 × ULN	90.68 (87.8–92.9)	35.66 (32.3–39.1)	49.5 (46.4–52.6)	84.6 (80.2–88.2)	0.632 (0.605–0.657)	3.55
4 × ULN	81.89 (78.3–84.9)	52.11 (48.5–55.6)	54.3 (50.9–57.7)	80.5 (76.8–83.8)	0.670 (0.644–0.695)	2.94
5 × ULN	77.95 (74.2–78.2)	58.60 (55.0–62.0)	56.7 (53.1–60.2)	79.2 (75.7–82.4)	0.683 (0.657–0.707)	2.73
5.5 × ULN (3010 μg/L)	75.22 (71.6–78.9)	61.72 (57.9–64.8)	57.7 (53.9–61.2)	78.2 (74.7–81.3)	0.727 (0.703–0.751)	2.70
6 × ULN	72.04 (68.0–75.6)	63.84 (60.3–67.1)	58.1 (54.3–61.7)	76.6 (73.2–79.7)	0.679 (0.654–0.704)	2.87

Abbreviations: CI, confidence interval; DD, D‐dimer; NPV, negative predictive value; PPV, positive predictive value; SE, sensitivity; SP, specificity; ULN, the upper limit of normal.

Considering different clinical scenarios, we analyzed various DD cutoff value based on the main diagnosis. As shown in Table 4, the surgical operation, aortic aneurysm, and serious infection subgroups had higher cutoff values, possibly due to a higher thrombosis burden. The application of the optimal DD cutoff value improved the specificity in the inpatient subgroups (Table 4).

**TABLE 4:** The optimal D‐dimer cutoff for the inpatient subgroups.

Inpatient subgroups (*N*)	Cutoff value	SE, %	SP, %	PPV, %	NPV, %	AUC
	(μg/L)	(95% CI)	(95%CI)	(95% CI)	(95% CI)	(95%CI)
Medical subgroup (1081)	3010	72.3	65.48	53.1	81.4	0.735
		(67.5–76.7)	(61.8–69.0)	(48.7–57.5)	(77.9–84.5)	(0.708–0.761)
Malignant tumor (417)	2600	77.05	61.97	61.3	77.5	0.732
		(70.3–82.9)	(55.4–68.2)	(54.7–67.6)	(70.9–83.3)	(0.686–0.773)
Serious infection (373)	4050	60.38	74.16	48.1	82.5	0.693
		(50.4–69.7)	(68.5–79.3)	(39.4–56.9)	(77.1–87.1)	(0.644–0.740)
Acute cerebrovascular disease (174)	3920	74.03	78.35	73.1	79.2	0.81
		(62.8–83.4)	(68.8–86.1)	(61.8–82.5)	(69.7–86.8)	(0.744–0.866)
Heart and kidney failure (85)	3600	50	84	29.4	92.6	0.595
		(18.7–81.3)	(73.7–91.4)	(10.3–56.0)	(83.7–97.6)	(0.483–0.700)
Aortic aneurysm (32)	4530	100	71.4	33.3	100	0.911
		(39.8–100.0)	(51.3–86.8)	(9.9–65.1)	(83.2–100.0)	(0.756–0.982)
Surgical subgroup (279)	4480	62.36	58.42	72.5	46.8	0.607
		(54.8–69.5)	(48.2–68.1)	(64.8–79.4)	(37.9–55.9)	(0.547–0.665)
Trauma (138)	3200	83.7	39.13	73.3	54.5	0.588
		(74.5–90.6)	(25.1–54.6)	(63.8–81.5)	(36.4–71.9)	(0.501–0.671)
Operation (141)	6090	41.86	81.82	78.3	47.4	0.611
		(31.3–53.0)	(69.1–90.9)	(63.6–89.1)	(37.0–57.9)	(0.526–0.692)

Abbreviations: DD, D‐dimer; NPV, negative predictive value; PPV, positive predictive value; SE, sensitivity; SP, specificity.

## 4 DISCUSSION

In this retrospective study at a single‐center in China, using an age‐adjusted DD cutoff values (age × 10 μg/L, for patients aged >50 years) increased the diagnostic efficacy for outpatients with suspected PE. For inpatients, we increased the DD value to at least 2 × ULN, which may reduce unnecessary CTPA tests in the elderly.

Several studies have explored methods to increase the SP of DD in older patients. Two possible approaches have been proposed, specifically, using age‐adjusted DD cutoff values or elevating the DD cutoff for all age groups. The ADJUST‐PE study^3^ firstly revealed that the age‐adjusted DD cutoff value may safely exclude the PE in older patients. And the 2019 PE guideline^7^ also recommended that using the age‐adjusted DD cutoff in outpatient by ELISA assay. However, our study first shows that the latex‐enhanced immunoturbidimetric method, which is more commonly used for the quantification of DD in China, functions similarly to the ELISA assay. DD values increase with age, making it difficult to rule out PE in older patients when the DD cutoff value does not take age into account. Many unnecessary CTPAs are therefore performed for older patients. Han et al.^14^ previously showed that using an age‐adjusted DD cutoff (age × 10, if age >50 years old) increased the proportion of Chinese outpatients for whom VTE could be safely ruled out. Our study demonstrated that the age‐adjusted strategy is suitable in Chinese outpatients tested by immunoturbidimetric method. And we also identified that it has significantly high diagnosis efficiency when compared the conventional cutoff value, with reducing the NNT. Douma et al.^8^ pointed out that the use of age‐adjusted cutoff produced a favorable effect on NNT (NNT = 2.2) in the outpatient by ELISA. Our study is similar to this result (NNT = 1.18) and better than it. A study has demonstrated that the INNOVANCE assay has a high negative predictive value (NPV) of 99.8% when using a new cutoff level of 1000 ng/mL, which is comparable to the ELISA assay.^3^, ^15^ Additionally, due to the high NPV (96.9%) of the age‐adjusted DD cutoff (age × 10, if age >50 years old) for outpatients in the emergency department, we recommended that an age‐adjusted D‐dimer cutoff value be used in conjunction with clinical probability assessment for outpatients from the Chinese population.

For inpatients, a previous study showed that when DD was measured using immunoturbidimetry, the DD level did not show a linear correlation with age, and an age‐adjusted DD cutoff value was not useful in the diagnostic algorithm for VTE.^16^ This result according to our study, the age‐adjusted cutoff performs poorly for inpatients, with SP as low as 13.34%. However, by raising the DD cutoff to at least 2 × ULN, NNT may reduce from 16.18 to 4.76 (*P* < 0.01). Some studies show that DD cutoff levels were at least twice as high compared to the conventional DD cutoff level of 500 μg/L (varying between 1000 and 4800 μg/L), yielding SE and SP values that varied between 63%–100% and 23%–84%, to discriminate COVID‐19 inpatient with and without PE.^17^ if we are raising the cutoff from 2ULN to 3010ug/L (about 5.5×ULN, the Youden's index) could rule out PE, it has better SP (61.72%) and poor NPV (78.2%). A recent study showed us the low DD cutoff (<1000 ng/mL), and PE can safely be ruled‐out without CTPA in COVID‐19 inpatients. In contrast, when we selected high DD cutoffs, based on the fact that we may correspond to a higher NPV, but not Youden's index, ensuring that PE can be safely ruled‐out without further testing,^18^ so the 2ULN maybe used the new threshold in this study, because of the high NPV (85.5%) and low NNT (4.76). A recent study takes the DD 3000 ng/mL cutoff as a predictive factor of PE in inpatients with COVID‐19 (OR = 7.494).^19^ This result is according to our result. Several previous studies found that the DD cutoff of 2400 μg/L was suitable for inpatients with suspected PE due to the comorbidity of community‐acquired pneumonia.^13^ For inpatients with comorbid acute aortic dissection and advanced lung cancer, the DD cutoff should be 4600 μg/L and 1140 μg/L, respectively, for PE.^2^, ^12^ Our study confirmed that DD cutoff values of 6090 μg/L, 4530 μg/L, 2600 μg/L, and 3200 μg/L are suitable for inpatients with suspected PE who recently underwent surgery or are comorbid with aortic aneurysm, trauma, and malignancy, respectively.

This study has several possible limitations. First, the present study was a single‐center retrospective study of DD levels measured with immunoturbidimetry in the Chinese population. Second, The DD cutoff of 2 × ULN for inpatients needs to be confirmed in a large‐scale multi‐center prospective study. Third, for the subgroup of inpatients, a multi‐factor analysis still needs to be conducted.

## 5 CONCLUSION

Using the immunoturbidimetric method to measure DD, an age‐adjusted DD cutoff (age × 10 μg/L, if aged >50 years) should be considered for outpatients with suspected PE. For inpatients, increasing the DD cutoff value to at least 2 × ULN yields the best test performance.

## AUTHOR CONTRIBUTIONS

Peng Liu and YingQun Ji contributed in original draft preparation, YingQun Ji and Haixu Yu in review and editing, YW and Wei Liu in article correction, and Lin Lin and YingQun Ji in supervision.

## CONFLICT OF INTEREST STATEMENT

The authors have disclosed that they do not have any potential conflict of interest.

## ETHICS STATEMENT

Ethical approval was given by the First Affiliated Hospital of DaLian Medical University Clinical Research Ethics Committee. The study was conducted according to the Declaration of Helsinki and its guidelines for Good Clinical Practice. (Ethical approval number:LCKY2017‐17).

## Data Availability

The derived data that were generated in the current study are available from the corresponding author upon reasonable request.
